# Blender tissue cartography: an intuitive tool for the analysis of dynamic 3D microscopy data

**DOI:** 10.1101/2025.02.04.636523

**Published:** 2025-07-14

**Authors:** Nikolas Claussen, Cécile Regis, Susan Wopat, Sebastian Streichan

**Affiliations:** 1 Department of Physics, University of California Santa Barbara, Santa Barbara, California 93106, USA; 2 Department of Bioengineering, University of California Santa Barbara, Santa Barbara, California 93106, USA

## Abstract

Understanding complex, three-dimensional tissues requires volumetric microscopy, but visualization, analysis, and processing of 3D image data can be challenging. Tissue cartography is an emerging method that exploits the sheet-like organization of many biological tissues. Extracting and cartographically projecting curved surfaces from volumetric image data turns 3D into 2D data, which is much easier to visualize, analyze, and computationally process. However, existing tissue cartography tools both require advanced coding expertise and are limited to specific tissue geometries. Here, we create an interactive, visual tool for tissue cartography within Blender, a popular 3D animation environment. blender_tissue_cartography (btc), opens tissue cartography to broad use via a user-friendly graphical interface, while harnessing powerful computer graphics algorithms to process a wide variety of biological shapes. An accompanying Python library allows faithful 3D measurements in 2D cartographic projections and the creation of custom analysis pipelines. btc batch-processes time-lapse data by propagating a cartographic projection from a single *key frame* to all other frames via surface-to-surface alignment. We demonstrate btc on diverse and complex tissue shapes from *Drosophila*, stem-cell-based organoids, *Arabidopsis*, and zebrafish. We believe our tool will open up a powerful set of analysis methods previously only accessible to specialists, enabling quantitative analysis of complex three-dimensional tissues and expanding our understanding of development.

Biological objects such as tissues, organs, or entire embryos and animals, are inherently three-dimensional (3D), requiring volumetric microscopy. This can make data storage, visualization, and quantitative analysis challenging. Tissue cartography takes advantage of the layered organization of many biological structures, such as epithelia, leaves, or tube-like visceral organs, to represent structures of interest as curved 2D surfaces embedded in 3D[1]. A tissue cartography workflow begins with the segmentation of a *surface of interest* (SOI) from 3D volumetric data (e.g. a *z*-stack from a confocal microscope), which is then cartographically *unwrapped* to a 2D plane. The volumetric image data can then be visualized and analyzed in the cartographic projection, reducing 3D- to 2D image analysis. This greatly facilitates tasks such as cell segmentation and tracking [2], measurement of tissue deformation [3], quantification of protein localization patterns [4], and *in-toto* visualization of curved objects. Tissue cartography has proven useful, notably in developmental biology, where tissues and organs dynamically change shape. Beyond their quantitative utility, cartographic projections enable the extraction of salient biological features, like tissue layers and organ geometry. Cartographic transformations allow “registering” multiple recordings (different samples or timepoints) to a common reference frame [5] and can thereby lay the ground for a quantitative understanding of development, as envisioned by D’Arcy Thompson in his classic “On Growth and Form” [6].

Existing tools such as for tissue cartography have enabled striking, quantitative insights into 3D morphogenesis [7, 2, 8]. This highlights the utility of the method. Yet, these implementations have shortcomings that impede widespread adoption and analysis of complex, dynamic geometries. Tools like LocalZProjector [9] parameterize surfaces via a *height function* and hence cannot handle fully 3D surfaces with “overhang”. The powerful 3D analysis software MorphoGraphX [10] has enabled sophisticated 3D analyses in the plant biology community. However, MorphoGraphX does not carry out cartographic projections at all and instead locally projects image intensity onto the vertices of a high-resolution surface mesh. This approach sidesteps cartographic projections but has certain limitations. In 3D, only part of a curved surface is visible at any time. Mesh-vertex-associated data is more complex and less flexible than cartographic projections (for example, it is not directly compatible with standard machine-learning-based image analysis software). These issues are especially relevant if the image cannot be easily segmented into cells. The ImSAnE [1] and TubULAR [3] software packages can carry out tissue cartography for more complex shapes. However, due to a lack of a graphical interface for editing surfaces and cartographic projections, and reliance on specialized MATLAB code, these tools are challenging for non-experts to use. Further, ImSAnE (developed in 2015) does not take advantage of sophisticated surface-processing algorithms developed by the computer vision community over the last decade (e.g. [11, 12]), while the more recent TubULAR is specialized to tube-like surfaces only.

Here, we present blender_tissue_cartography (btc), an add-on to the popular, open-source 3D editor Blender [13] and an accompanying Python library. The btc suite makes the analysis of dynamic, highly curved surfaces via tissue cartography accessible to non-experts while adding new capabilities, by taking advantage of high-quality tools developed by the computer graphics and 3D animation community. btc features a unified, modular design, a graphical user interface, and a general-purpose pipeline for the analysis of dynamic datasets. btc comes with detailed documentation, tutorials, and a set of template analysis pipelines.

## Results

### Tissue cartography workflow in btc

**Figure 1:**
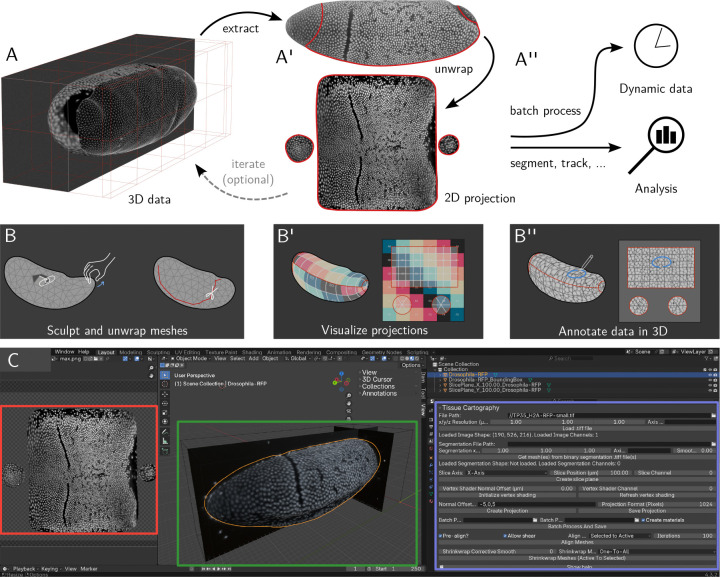
btc for powerful and user-friendly 3D analysis workflows. A-A” (A) 3D image data image (*Drosophila* blastoderm with fluorescently labeled nuclei, anterior left, ventral down). (A’) The extracted curved 2D surface of interest is represented by a polygonal mesh. Surface unwrapping (UV mapping) allows the projection of 3D image data into 2D. After inspection of the results, meshes and UV maps can be rapidly edited to improve the quality of 2D projections. (A”) 2D projections can be used for *in toto* visualization or downstream analyses such as cell segmentation. Batch processing tools allow the processing of dynamic datasets (see Fig. 3). Modular design allows switching out the tool used in each pipeline step. **B-B”** Interactive 3D data processing in Blender’s graphical user interface. (B) Surface sculpting for fixing segmentation errors. Blender’s UV editor combines powerful cartographic algorithms with graphical user input (e.g. placement of cartographic cuts). (B’) Cartographic projections and their distortion can be visualized in 3D, for instance by mapping a color grid onto the mesh surface. (B”) Projected data can be visualized and annotated in 3D, with results saved to 2D projections. **C** Blender user interface. Cartographic projection (red), surface of interest with 3D data visualized by ortho-slices and projected mesh intensity (green), btc add-on toolset for loading and processing 3D data (blue).

Tissue cartography generally proceeds in the following steps (Fig. 1A–A”):

1. **Extraction:** Detect the surface of interest in volumetric image data, typically via 3D image *segmentation*, and convert the result to a surface mesh (*meshing*).
2. **Unwrapping:** Unwrap the 3D surface to a 2D cartographic plane.
3. **Image projection:** Project 3D image data into 2D.
4. **Batch processing:** Batch process e.g. the frames of a time-lapse recording.
5. **Analysis and visualization:** Measure quantities of interest in the 2D projection, accounting for the surface’s 3D curvature.

We now describe how these steps are implemented in btc. btc comprises two tools:

1. **The** btc **Blender add-on** allows users to carry out tissue cartography within a graphical user interface without requiring any coding (Fig. 1C). The add-on makes Blender’s sophisticated toolset (surface sculpting, unwrapping, and annotation, Fig. 1B–B”) available for the analyses of 3D image datasets. 3D data can be visualized throughout the process, greatly facilitating, for instance, choosing a suitable cartographic projection.
2. **The** btc **Python library** is an open-source, cross-platform Python package available for installation on pip and allows advanced users to create custom and automatized analysis pipelines. Each pipeline step terminates in a single file of standardized type (e.g. a .obj mesh file). Due to this modular design, the tool used for each step can be easily switched out. This is essential as scientific imaging data is often highly diverse. Provided tutorial Jupyter notebooks can serve as starting points for specialized pipelines.

Comprehensive documentation (including algorithm details) for both the library and the add-on, tutorials, and a set of template analysis pipelines can be found online. A 2-minute video demonstration is also available.

### Extraction: segmentation and meshing

To extract the surface of interest, the volumetric image is first segmented, detecting either (a) the voxels that are part of the SOI or (b) the voxels that are part of the solid object whose boundary is the SOI (see examples in Fig. 4A–A”). btc provides an interface to carry out this binary segmentation using the popular machine learning software ilastik [14] and via level set algorithms [15]. Next, the resulting binary mask is converted into a surface mesh using Poisson reconstruction [16] or the marching cubes algorithm (depending on the option (a) or (b) above). Throughout btc, surfaces are represented as polygonal meshes (*V*, *T*), where *V* is a set of vertex positions, and *T* is a set of polygonal faces that stitch the vertices together to form a surface. btc contains a variety of functions for mesh quality improvement (remeshing), smoothing, and processing.

Within Blender, the resulting meshes can be inspected and potential segmentation errors can be fixed using Blender‘s 3D editing tools (Fig. 1B). The volumetric image data can also be loaded into Blender, allowing the user to sculpt the mesh while visualizing the image data in 3D or projected onto the mesh surface.

### Surface unwrapping and image projection

Now, the mesh can be cartographically unwrapped to a 2D plane (Fig. 1A’). This is known as *UV mapping* in the graphics community as the 2D plane coordinates are conventionally denoted *u*, *v* (versus *x*, *y*, *z* for the 3D coordinates). Blender possesses a powerful graphical UV editor that implements a variety of cartographic algorithms, from standard axial, spherical, and cylindrical projections to powerful, state-of-the-art algorithms like SLIM [11] which can unwrap even complex surfaces with minimal distortion. Additionally, Blender allows the user to define the location of *seams* (cartographic cuts, (Fig. 1B), manually fine-tune the results, and visualize the projection (Fig. 1B’).

The resulting UV map of a surface (*V*, *T*) is represented in a standardized fashion as a set of 2D UV vertices in the unit UV square *V*_uv_ ⊂ [0, 1]^2^, and a set of UV faces *T*_uv_ that are in one-to-one correspondence with the 3D faces. This defines how the 3D surface is mapped to the plane. The mesh and its UV map can be exported to or loaded from an .obj mesh.

Given the SOI mesh with a UV map, btc uses an interpolation algorithm to project voxel intensities from the volumetric data onto the 2D UV plane. We refer to the result as a *2D projection* (Fig. 1A’, bottom). The interpolation scheme is designed so that the resolution of the 2D projection does not depend on that of the mesh, i.e. a coarse, lightweight mesh can still be used for high-resolution projections. btc features a second, simpler projection algorithm that evaluates 3D image intensities at mesh vertices. This vertex-shader algorithm does not require a UV map and can be recomputed rapidly, allowing the user to visualize image intensities on the mesh surface while sculpting or unwrapping it.

### Analysis and visualization

btc saves the 2D projection (and 3D positions for each pixel in the projection) both as .tif stacks for 2D visualization and analysis, as well as Blender textures for visualization on the 3D mesh. This allows not only rendering high-quality figures but also rapid, iterative improvements of the mesh and its UV map. For instance, the user can adjust the placement of cartographic seams based on the image textures, e.g. focusing on a region of particular interest. Further, Blender‘s “texture paint” tools can annotate data in 3D, with results (e.g. cell labels) saved on top of 2D projections for downstream analysis (Fig. 1B”).

For quantitative analysis, btc provides a suite for measuring and correcting for cartographic distortion and mapping quantities measured in 2D back (like cell tracks or outlines)back to 3D. For instance, these tools can correctly compute the area of a cell in a 2D projection, even if the unwrapping introduces local area distortion. All required quantities can be robustly computed based on the triangular mesh structure [17].

We also provide a simple implementation of vector calculus on curved surfaces, for instance, to analyze morphogenetic tissue flows obtained from particle image velocimetry (PIV) or cell tracking in 2D projections [4, 2]. In brief, vector and tensor fields on a surface are represented by their Cartesian 3D coordinates and then separated into tangential and normal components. Using standard finite-element operators on the mesh, gradients can be calculated component-wise and combined to form the curved-surface generalizations of familiar operators like div, grad, and curl [18], and approach accessible to non-experts in differential geometry.

### Multilayer projections

**Figure 2:**
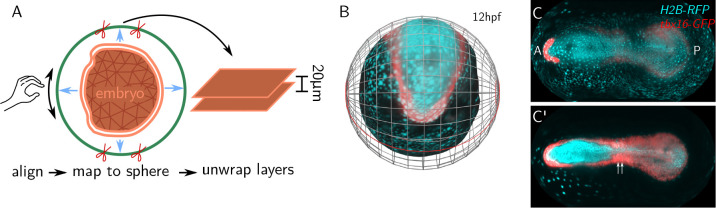
Extraction of multiple tissue layers. **A** A sphere (green) is aligned to a zebrafish embryo (orange mesh) extracted from an *in toto* lightsheet recording. The embryo’s surface is projected to the sphere, which is then unwrapped. Rotation of the sphere allows visual alignment of the cartographic projection with anatomical features like the AP axis. btc allows extracting multiple tissue layers within a common cartographic projection. **B** Surface of a zebrafish embryo at 12hpf (neurolation), together with sphere used for cartographic projection. Fluorescent intensities of 3D data is projected onto the embryo surface (cyan: H2B-RFP (nuclei), red: tbx16-GFP [19] (mesoderm)). Locations of cartographic cuts are highlighted in red. **C-C’** Fluorescent intensities cartographically projected into 2D, at the surface of the embryo (C) and 20*μ*m below (C’). Mesoderm, marked by tbx16-expression, is localized in the deeper layer and shows the formation of somites (white arrows). The region of high tbx16 expression in the anterior is the prechordal plate.

btc can create multi-layer projections, analogous to peeling off the layers of an onion. Each layer is offset inwards or outwards by a desired distance from the SOI, using the local surface normals. Fig. 2 showcases multilayer projections of an *in toto* recording of 12hpf (hours post fertilization) zebrafish embryo. The embryo is unwrapped using a spherical projection (Figs. 2A–B). Using the vertex shader, the user can visualize the image intensity while rotating the sphere to visually align the equator of the spherical projection with the anterior-posterior axis of the animal. Figs. 2C–C’ shows two layer projections, at the surface of the embryo and 20*μ*m inwards, where the mesoderm and the forming somites are visible.

The online video demonstration shows the above steps for the example *Drosophila* dataset from Fig. 1.

### Batch processing and dynamic datasets

The workflow described so far applies to a single surface of interest. Often, however, we are interested in dynamic datasets (movies), or multiple recordings of similarly shaped objects (e.g. multiple *Drosophila* blastoderm stained with different markers[20]). In btc, time-lapse datasets are represented frame-by-frame (one surface mesh and volumetric image file per timepoint). Such collections of SOIs can be batch-processed via the btc add-on or Python library.

It is highly desirable to use “the same” UV map for each time point, both to facilitate comparison of the 2D projection across time points or samples and because it would be cumbersome to manually define a UV map each time. There are two possible strategies.

The first possibility is to compute a UV map for each mesh individually, but using a consistent algorithmic procedure. This approach works well for simple shapes that can be unwrapped by axis, cylindrical, or spherical projections which can be batch-computed in Blender. However, for more complex shapes (in particular, if unwrapping requires seams), choosing or designing a suitable algorithm can require significant computer vision expertise and, crucially, cannot be done graphically and interactively.

**Figure 3:**
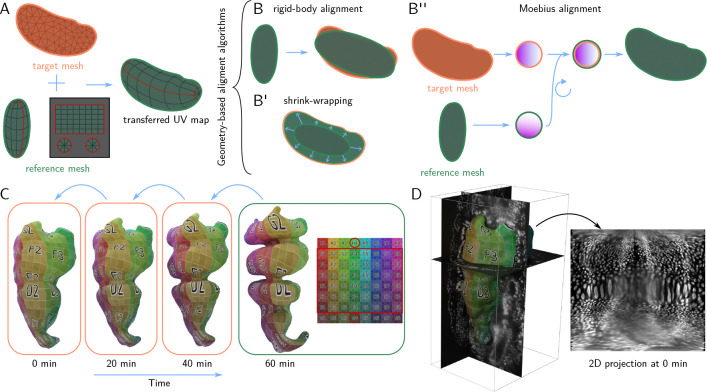
Batch-processing and dynamic data via UV surface-surface alignment. **A** Transferring a UV map to the orange target mesh from the green reference mesh via surface-to-surface alignment: the reference mesh is deformed to match the shape of the target mesh while preserving its UV map. **B-B”** Surface-to-surface alignment algorithms: rigid-body alignment, shrink-wrapping (smoothed closest-point projection), and Moebius alignment (see text). All algorithms are based on the shape of the surface only. **C** UV transfer for a highly dynamic biological surface. Mesh of the embryonic *Drosophila* midgut extracted from live deep-tissue *in-toto* imaging (data from [2]). The complex shape of the final timepoint is unwrapped in Blender and the UV map is iteratively transferred to all other timepoints via shrink-wrapping. **D** The extracted surface at timepoint 0 together with 3D data (fluorescently marker nuclei) and 2D projection.

Instead, in btc, the user defines a UV map for a selected *reference mesh* – e.g. the first or last frame of a movie, or an “idealized” version of the imaged shapes – using Blender‘s graphical UV editor. The reference mesh is then algorithmically mapped onto the meshes of the remaining time points (the *target meshes*), a process that we refer to as *surface-to-surface alignment* (Fig. 3A). This allows *transferring the UV map* from the reference mesh to all other meshes and hence creates coherent 2D projections across time points or samples. In particular, the layout of the 2D projection in the UV square does not change. For the case of multiple samples, surface-to-surface alignment allows combining recordings of e.g. different markers into a single “atlas” [20]. Surface-to-surface registration has received significant attention in the computer graphics community. In btc, we implement three algorithms, all based purely on surface geometry (i.e. they make no use of the 3D image data):

1. **Rigid-body alignment.** The reference mesh is translated, rotated, and scaled to match the target mesh (Fig. 3B).
2. **Closest-point projection (shrink-wrapping).** The reference mesh is first rigidly aligned to the target, and then each vertex of the reference mesh is projected to the closest point on the target surface (Fig. 3B’). This operation is combined with smoothing to remove “creases”.
3. **Moebius alignment** [21]. Both reference and target mesh are mapped to a reference shape (a disk, a punctured disk, or a sphere, depending on the surface topology) in a way that preserves triangle angles. Mathematically, this *conformal map*^1^ to the reference shape is guaranteed to be almost unique, up to a small set of *Moebius transformations* (e.g. rotations) which are chosen to best align the geometry of the reference and target mesh (Fig. 3B”).

For dynamic datasets, surface-to-surface registration can done iteratively (i.e. first map reference to the first frame, then the result to the second frame, etc.), significantly reducing its difficulty (Fig. 3C).

Thanks to btc‘s extensible Python interface, external libraries such as PyFM (implementing so-called “functional maps” [24]) can also be used. The above algorithms are fully automatic and do not require the user to specify point-to-point correspondences between reference and target mesh, which is time-consuming and can introduce bias in the absence of clear shapes enabling interest point placement. The Moebius algorithm is highly robust and will find a surface-to-surface map even between two strongly deformed meshes [21].

We validated btc‘s approach to time-lapse data on an example of a highly dynamic surface, the embryonic *Drosophila* midgut (Fig. 3C–D, data from Ref. [2]). We find that it is best to choose the time point with the most complicated shape (here, the final one), as the reference mesh. The reference mesh is unwrapped in Blender, along a user-defined seam that follows the long axis of the organ. The UV map is then iteratively transferred to the remaining transferred using the closest-point projection algorithm (Fig. 3C), allowing computing projections across all time points (Fig. 3D). The algorithm faithfully transfers the UV map across time points, with only minor degradation close to mesh seams.

### btc can process diverse and complex shapes

**Figure 4:**
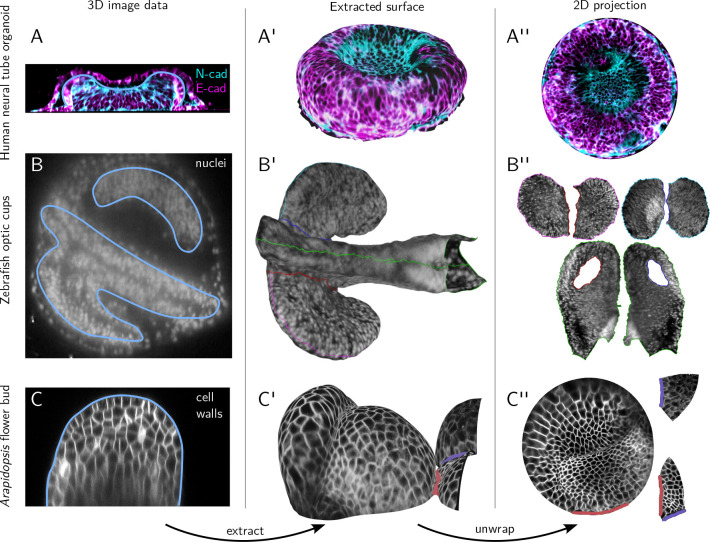
btc processes complex and diverse shapes across biological contexts. A-A” Confocal *z*-stack of a human neural tube organoid [25], stained for neural- and epithelial cadherin. (A) Cross-section of 3D image data, substrate at bottom. The extracted organoid develops within a lumen, visible outside the blue contour which indicates the extracted surface. (A’) 3D rendering of the extracted surfaces. (A”) Corresponding 2D projection. **B-B”** Lightsheet recording of a 16hpf zebrafish embryo, with fluorescently marked nuclei. Anterior left, ventral view. (B) Cross section of 3D data. (B’) Extracted surfaces with seams of the UV map marked in color. (B”) Corresponding 2D projection with multiple patches corresponding to the ventral and dorsal sides of the optic vesicles and the lateral sides of the neural tube. **C-C”** Confocal *z*-stack of *Arabidopsis thaliana* flower buds with a membrane marker. (C) Cross-section of 3D image data. (C’) Extracted surface. (C”) 2D projection. Cell outlines can be used e.g. for segmentation. **D-D”** Quantitative analysis using cartographic projections. (D) Area distortion of UV map from C”. (D’-D”) Segmentation of 2D projected *Arabidopsis thaliana* data, colored by (D’) uncorrected 2D cell areas and (D”) cartographically corrected, 3D cell areas.

Next, we demonstrate the applicability of btc to complex and diverse shapes from a range of biological contexts (Fig. 4, data sources described in Materials & Methods). Our first example is a confocal *z*-stack of a human neural tube organoid grown on a micropatterned substrate [25] (Fig. 4A–C). Even for this relatively simple shape, tissue cartography offers clear advantages over a maximum *z*-projection, as well as height-map-based approaches like LocalZProjector [9], both of which are unable to follow the curved apical surface of the organoid due to overhangs. Note also that btc supports arbitrary multichannel data (here, neural and epithelial cadherin stainings).

Next, we extracted the anterior neural tube and developing optic vesicles [26] from an *in-toto* light-sheet recording of a 16hpf zebrafish embryo (Fig. 4A’–C’). This complex shape is beyond the reach of the ImSAnE or TubULAR software, but can be easily unwrapped along seams following morphological landmarks, selected graphically within Blender (Fig. 4B’). Segmenting out this shape from 3D data (in particular, separating it from the surrounding EVL tissue layer), is also greatly streamlined by Blender’s sculpting and modeling tools. Our last example are *Arapidospis thaliana* flower buds (Fig. 4A”–B”).

### Quantitative analysis in cartographic projections with the btc Python library

**Figure 5:**
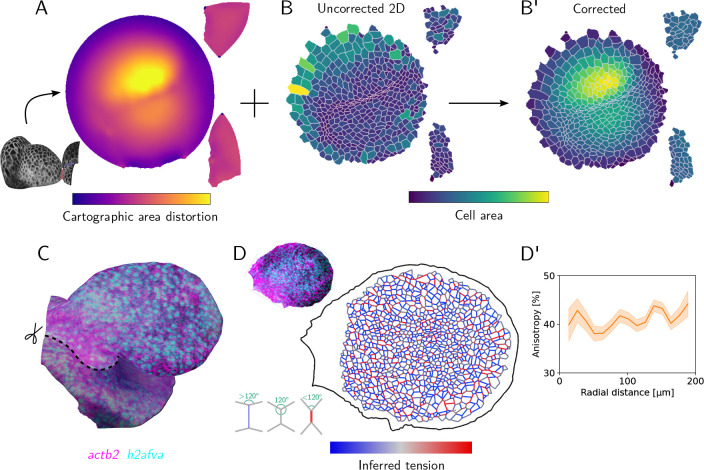
Quantitative analysis of data on curved surfaces with the btc Python library. **A** Area distortion of UV map for the *Arabidopsis thaliana* data (inset, Fig. 4C”). **B-B’**(D’-D”) Segmentation of 2D projections, colored by (B) 2D cell areas and (B’) cartographically corrected, 3D cell areas. **C** 3D mesh of the developing optic zebrafish optic cup at 16hpf (see Fig. 4B), dorsal-anterior view (cyan: h2afva (nuclei), magenta: actb2 (membrane)). Attachment to the neural tube visible on the left. Analyzed portion of the surface in bright. **D-D’** Analysis of cell mechanics on the apical surface of the optic cup. Projected nuclear and membrane signal (top inset) was used to segment out cells and infer relative interfacial tensions on cell-cell interfaces from cell geometry (bottom inset). (D’) Tension anisotropy (deviatoric part of tricellular vertex stress tensor [8]) shows no clear center-to-margin pattern, indicating the absence of a tension cable around the optic cup margin.

Cartographic projections are not only useful for visualization but also for quantitative analyses: image analysis in 2D is significantly easier than in 3D. However, like a map of the globe, cartographic projection introduce distortion. The btc Python library contains a set of tools for mathematically correcting cartographic distortion and mapping points in the projection back into 3D. Tools for shape analysis are also included. In Fig. 5, we present two examples. Fig. 5B shows a cell segmentation of the *Arabidposis* flower buds from Fig. 4C. Correcting for the area distortion of the cartographic projection (Fig. 5A) allows bonafide measurements of cell areas (Fig. 5B’). Fig. 5C provides a closer look at the zebrafish optic vesicle (Fig. 4B). Segmenting out the cells on the dorsal side of the cup (which eventually folds in to form the eye cavity) allows carrying out *mechanical inference* [8] (Fig. 4D). Under the assumption that mechanical stresses are concentrated along cell-cell interfaces, the interfacial tension can be inferred from the geometry of cell interfaces (Fig. 4D, bottom inset). To do this, tricellular vertex positions are detected in the 2D segmentation and then mapped back to 3D to correctly measure the cell geometry. We find no clear pattern at this developmental stage (Fig. 5D’); in particular, there is no tension cable around the margin of the optic cup as might be expected in a “purse string” mechanism for folding.

## Discussion

Here, we presented blender_tissue_cartography (btc), a software suite for analyzing dynamic 3D microscopy data via the 3D editor Blender. Tissue cartography extracts and cartographically projects surface-like structures from volumetric data, reducing 3D to 2D data analysis and enabling quantitative insights into morphogenetic dynamics during animal and plant development. Thanks to btc‘s graphical user interface, suitable cartographic projections can be iteratively designed for new and complicated geometries. Our pipeline for dynamic movies preserves the ability of the user to graphically define their UV map and is applicable without modification across a wide range of datasets.

We have demonstrated btc on a variety of static and dynamic datasets with complex geometries from four different model organisms, notably on *in-toto* recordings of the zebrafish embryo. The round yolk mass of the zebrafish embryo has made visualization of its anteroposterior axis in its entirety challenging. Typically, embryos must be fixed, dissociated from their yolk, and flat-mounted to provide a full view of the embryonic body [27]. The cartographic projections shown in Fig. 2 now make this feasible in a living embryo, enabling unhindered, dynamic observation of somitogenesis and neurulation.

In particular for such dynamic data, as well as quantitative comparison across samples, registering multiple images into the same reference frame is essential [5]. This task is greatly facilitated by cartographic projections. Using Blender’s UV editor, the user can map anatomically salient features (like the anterior pole of the zebrafish) to defined cartographic positions.

In our experience, btc has proven user-friendly and easy to learn, with second-year undergraduates and scientists with no computer programming experience able to analyze the data of their interest. We believe btc will make the analysis of dynamic 3D surfaces accessible to a broad audience and thus further our understanding of morphogenesis. Approaching development and biological shape via cartographic transformations was originally proposed by D’Arcy Thompson in 1917, and we hope that our work will help connect this vision with the striking microscopy data of the 21^st^ century.

## Methods

### btc add-on and Python library

The btc Python library is built using the standard Python scientific computing software stack [28, 29, 30] and uses a lightweight mesh data representation inspired by the .obj mesh file format. The geometry-processing library igl is used as the main geometry backend [31]. MeshLab [32] is used for certain advanced (re)meshing operations. The source code is available on GitHub. The Blender add-on is based on the btc library code with adaptions to interface with the Blender API and is also available on GitHub.

### Data sources

The *Drosophila* blastoderm in Fig. 1A shows a multiview light-sheet microscope recording of a *+;+;H2A:RFP* embryo at stage 6 from Ref. [20]. Fig. 3C–D shows the embryonic *Drosophila* midgut extracted from a multiview lightsheet microscope recording of a *Hand:GFP;+;Hist:GFP* embryo at stage 15 from Ref. [2]. Fig. 4A–C shows a confocal microscope recording of a human neural tube organoid stained for N-cadherin and E-cadherin at 48 hours post BMP addition (organoid protocol initiation) from Ref. [25]. Fig. 4A”–C” shows a confocal recording of *Arabidposis thaliana* flower buds with plasma membrane marker *pUBQ10::29–1-GFP* [33], shared Dr. An Yan. Permission to display the data was obtained from the respective authors of the respective publications. The zebrafish datasets in Figs. 2, 4B, and 5C were recorded for this manuscript (see next section).

### Zebrafish microscopy

Adult zebrafish of the AB/TU background were used for experiments and housed and bred using standard conditions [34]. All experiments were performed with institutional approval from the University of California, Santa Barbara. Zebrafish embryos were mounted in agarose and imaged in a multi-view light-sheet microscope [35], as described previously [36]. Multi-view data was registered and fused as described previously [36, 37] to obtain volumetric image data with isotropic resolution of 0.26*μ*m. We used the following fluorescent reporter lines: *Tg(tbx16:RFP)* [19], microinjected with H2B-RFP mRNA (Fig. 2), *Tg(h2afva:h2afva-GFP)* (Fig. 4), and *Tg(h2afva:h2afva-GFP)kca66; Tg(actb2:memCherry)hm29* [38] (Fig. 5). For mRNA injection, H2B-RFP mRNA (a gift from the Wallingford lab) was synthesized from NotI-linearized pCS2+-H2B-RFP plasmid using the SP6 mMessage mMachine kit (Invitrogen). A total of 50 pg of H2B-RFP was microinjected into single-cell embryos with pulled glass needles.

### Data processing

Data was processed following the pipeline described in the main text methods section. In brief, all volumetric datasets were segmented using ilastik with additional post-processing via level set methods for the zebrafish dataset. The volumetric segmentations were converted to meshes using the btc Blender add-on, and remeshed for improved mesh quality where necessary using either Blender or MeshLab. For the embryonic *Drosophila* midgut data, we used surface meshes generated in Ref. [2]. UV maps were generated using the “Minimum Stretch” and “Angle Based Flattening” algorithms in Blender [11] after graphical seam placement. To obtain a cylinder-like projection for the embryonic midgut data, we used the “Pin” and “Align” tools of the UV editor to ensure straight seams. All 2D projections and 3D renderings were created in Blender, using the btc add-on. Cell segmentations were carried out using Tissue Analyzer [39], and mechanical inference was carried out following the method of Ref. [8] and using the codebase Ref. [40].
